# Volumetric Control vs. Pneumatic Pressure: A Comparative Analysis of Extrusion in 3D Bioprinting

**DOI:** 10.3390/mi17050521

**Published:** 2026-04-24

**Authors:** Doru-Daniel Cristea, Eduard Liciu, Andreea Trifan, Corneliu Bălan

**Affiliations:** 1 3D Printing Laboratory, Center of Innovation and e-Health, Carol Davila University of Medicine and Pharmacy, 020021 Bucharest, Romania; andreea.trifan@umfcd.ro; 2 REOROM Laboratory, Hydraulics Department, Power Engineering Faculty, National University of Science and Technology Politehnica Bucharest, 060042 Bucharest, Romania; corneliu.balan@upb.ro; 3 Faculty of Chemical Engineering and Biotechnologies, University Politehnica of Bucharest, 011061 Bucharest, Romania

**Keywords:** 3D bioprinting, DIW, biomaterial ink, quality assessment

## Abstract

Extrusion-based bioprinting faces significant challenges in achieving the shape fidelity and internal porosity necessary for cell viability, often hindered by subjective assessment methods. This study investigated the relationship between rheological properties and print quality using a natural polymer biomaterial ink composed of 12% gelatin, 5% alginate, and 1% carboxymethylcellulose. We conducted a comparative analysis between traditional pneumatic systems and screw-driven volumetric extrusion, utilizing a suite of quantitative metrics: Spreading Ratio (SR), Printability Index ($P_{r}$), Uniformity Ratio (UF), Collapse Angle (θ), and evaluated porosity. Our results demonstrate that the screw-driven system’s positive displacement mechanism provides superior control over filament morphology by enabling precise volumetric modulation. While the pneumatic system exhibited a high SR of 1.82 and the lowest porosity at 59.92%, the screw-driven system allowed for “under-extrusion” to compensate for viscoelastic die swell. Reducing the flow rate to 50% in the screw system lowered the SR to 1.09, nearly matching the nozzle diameter, and increased porosity to 76.46%. Furthermore, the screw-driven system achieved an ideal $P_{r}$ of 1.0, whereas the pneumatic system produced distorted, rounded pores with a $P_{r}$ of 1.57. The findings indicate that screw-driven extruders can decouple line complex rheology from the printing process, allowing for finer spatial resolution and better pore interconnectivity.

## 1. Introduction

Additive manufacturing has emerged as a transformative technology for regenerative medicine [1], enabling the fabrication of complex [2], patient-specific tissue constructs [3] with spatial control over cellular distribution and biomaterial composition. Extrusion-based bioprinting has established itself as a leading modality [4], driven by the urgent clinical need to fabricate functional tissue substitutes that accurately mimic the complex, hierarchical architecture of native human tissues [5]. At this scale, the ability to resolve micro-details is not merely a geometric goal but a requirement for replicating the intricate micro-environments that govern cellular behavior. According to the consensus definition proposed by Groll et al., [6] the term “bioink” should be reserved for cell-containing formulations used for biofabrication, whereas acellular printable formulations are more appropriately referred to as “biomaterial inks”. The present study focuses on an acellular biomaterial ink, while the broader context of bioink development remains directly relevant for future cell-laden applications. The clinical motivation for this work is multifaceted: printed constructs must match specific anatomical geometries [7] derived from patient imaging data (CT or MRI scans), particularly for load-bearing tissues like cartilage or aesthetic replacements like ears and skin [5]. Small geometric errors in early print layers propagate as fabrication progresses, causing structural failure [8]. Critically, printed scaffolds must maintain open, perfusable microchannels for oxygen and nutrient diffusion [9]. The preservation of high-fidelity micro-detail, specifically the sharpness of pore corners and the consistency of channel diameters, represents a primary technical bottleneck in ensuring construct viability. If these pores close due to filament fusion or spreading, cells deep within the construct may die. Current bioink [6] development relies heavily on time-consuming, resource-intensive trial-and-error optimization with subjective visual assessment, a particularly problematic approach when using expensive biological materials such as growth factors or patient-specific cells [3]. There is an urgent need for standardized, quantitative methods to assess printability and predict print quality, enabling faster optimization and more confident clinical translation [10].

Extrusion-based bioprinting has become the preferred modality for many tissue engineering applications for several well-established reasons. Extrusion systems operate across the broadest range of material viscosities, processing fluids from 30 mPa·s to over 6 × 10^7^ mPa·s, far exceeding the 3.5–12 mPa·s capability of inkjet systems [5]. This viscosity range enables the processing of biomaterial inks such as high-concentration hydrogels, pastes, and composites that provide superior mechanical support [11]. For tissue engineering, extrusion systems can achieve physiologically relevant cell densities exceeding 10^8^ cells/mL, substantially higher than the 10^6^ cells/mL feasible with inkjet printing before nozzle clogging occurs [12]. The continuous filament deposition of extrusion printing facilitates layer-by-layer construction of large, self-supporting 3D structures without extensive sacrificial support materials [9]. The inherent modularity of extrusion platforms, with multiple independently controlled printheads [13], enables rapid material switching within a single construct, facilitating the fabrication of heterogeneous tissues. Furthermore, extrusion technology is more accessible and cost-effective [14] than laser-assisted or stereolithography approaches [15], having evolved from standard fused deposition modelling (FDM) hardware [16]. Soft tissue regeneration, including skin, vascular networks, cardiac patches, and neural conduits and hard tissue repair for bone defects and cartilage replacements represent the primary applications driving extrusion bioprinting research [17].

In the field of extrusion bioprinting, there are three primary design configurations for the extrusion system: pneumatic, piston, and screw-driven [10]. The most widespread approach utilizes a pneumatic syringe filled with biomaterial ink to deposit material onto a substrate. While this system is the most cost-effective and straightforward, it faces significant limitations [18]. Specifically, it struggles with high-viscosity materials and offers poor flow control. Furthermore, the compressible nature of air and the compliance of the air lines cause considerable hysteresis, leading to material oozing and inconsistent starts or stops [19].

Screw-driven systems offer significant advantages over their pneumatic counterparts. While they excel at processing highly viscous gels and pastes [20], their primary strength lies in their operation as positive displacement pumps [21]. This mechanism enables precise volumetric control, as the flow rate remains linearly proportional to the motor’s rotational speed [22]. By directly modulating motor rotation, the extrusion volume is adjusted in a predictable fashion, facilitating sharp “start-stop” transitions and dynamic flow control. This eliminates hysteresis and lag characteristic of pneumatic systems, which are typically affected by air compressibility [12].

In pneumatic extrusion, the non-Newtonian nature of bioinks—particularly their viscoelastic recovery and ‘elastic memory’—often leads to over-swelling and spreading because the applied air pressure cannot relax instantaneously when travel speed changes. This hysteresis causes delayed starts, oozing and irregular filament diameters [12], all of which reduce geometric fidelity. By contrast, screw-driven extruders operate as positive-displacement pumps, where the volumetric flow rate is linearly coupled to motor rotation. This mechanically ‘locks’ the extruded volume to the commanded toolpath, reducing the influence of transient pressure fluctuations and better preserving filament geometry and pore shape [10].

This study evaluates the comparative print quality and micro fidelity achievable by pneumatic and screw-driven extrusion systems through a standardized benchmarking suite [23]. We aim to demonstrate that the positive displacement mechanism of screw-driven systems can resolve finer detail by decoupling volumetric output from travel speed, a critical factor for maintaining the intricate internal architectures required in tissue engineering. In extrusion-based bioprinting, “quality” is typically defined as a combination of printability (the ability to extrude a stable, continuous filament) and shape fidelity (the agreement between the printed geometry and the digital design) [3]. Since comprehensive rheological characterization requires specialized instrumentation that is often inaccessible, researchers frequently rely on in situ, printer-based assessments [24]. These methods utilize the bioprinter’s optical systems and image analysis software (e.g., Fiji/ImageJ) to quantify post-deposition geometry [4]. Such tests are advantageous due to their efficiency and their ability to directly probe common failure modes, including spreading, fusion, sagging, and flow instabilities [25].

Our benchmarking workflow begins with an extrudability screen (filament drop test) to verify continuous fiber formation, followed by 2D grid printing to calculate the Printability Index. Post-deposition spreading is quantified via the Spreading Ratio, while filament fusion is evaluated by measuring the merging threshold across decreasing gap widths. To assess structural integrity, a filament collapse test across increasing spans is used to determine the collapse angle. Furthermore, line prints allow for the quantification of extrusion homogeneity through the Uniformity Ratio. In this work, we define and implement these core metrics before extending the analysis to include layer-wise evaluations and overhang quality assessments specifically tailored for biomaterial ink extrusion.

## 2. Materials and Methods

### 2.1. Extrusion Systems

Two distinct hardware platforms were employed to evaluate the comparative performance of the extrusion mechanisms. The pneumatic benchmarking was conducted using a commercial CELLINK Bio X bioprinter (CELLINK, Gothenburg, Sweden), representing the industry standard for air-driven extrusion. In contrast, the screw-driven assessments were performed on a custom-engineered platform integrated with an LNM0.01CC single screw pump (Wuhan Ronice Trading Co., Ltd., Wuhan, China). This positive displacement system features an adjustable flow rate ranging from 0.33 to 1.2 mL/min, with a precise volumetric output of approximately 0.01 mL per motor revolution. The customized platform uses Klipper firmware (version 0.12) [26,27] for motor control.

To ensure the theoretical dosing of 0.01 mL per revolution (10 mm^3^/rev) translated accurately into physical extrusion, the system was calibrated within the Klipper firmware environment. Rather than using standard linear extrusion based on a 1.75 mm filament diameter (standard for FDM printers) [25], a volumetric extrusion workflow was implemented to improve the intuitiveness and precision of the control logic.

The calibration process involved a “diameter trick” within the slicer settings: by setting the theoretical filament diameter to 1.12838 mm, the cross-sectional area becomes exactly 1 mm^2^. Consequently, every 1 mm of extrusion commanded in the G-code (E value) represents exactly 1 mm^3^ of material volume. Correspondingly, the Klipper (version 0.12) “rotation_distance” was configured to a value of 10, aligning a 10 mm G-code command (10 mm^3^) with exactly one full motor revolution. This configuration allows for direct volumetric control and eliminates the need for complex conversion factors during the benchmarking of printability and shape fidelity.

All constructs were printed at room temperature (25 °C). Given the short printing times (≤45 s per construct), thermal gradients within the biomaterial ink were negligible and no additional temperature-control hardware was required during the benchmarking process.

The pneumatic pressure (75 kPa) was selected as the minimum threshold necessary to initiate a stable, continuous extrusion (Minimum Extrusion Pressure). For the screw-driven extruder, a sweep from 100% down to 50% extrusion factor was implemented to map the transition from nominal volumetric dosing to deliberate under-extrusion, identifying the range in which die swell is compensated while filament integrity and pore geometry are maintained, as shown in Table 1.

### 2.2. Biomaterial Ink Formulation

Sodium alginate (CAS 9005-38-3), porcine gelatin (CAS 9000-70-8), calcium chloride (CaCl_2_, CAS 10043-52-4) and glutaraldehyde (0.5% solution, CAS 111-30-8) were purchased from Sigma-Aldrich (St. Louis, MO, USA). Citric acid (3% solution) was used as additional crosslinking agent and carboxymethylcellulose (CAS 9004-32-4, DanidaCHEM, Bucharest, Romania) were acquired.

To prepare 50 mL of biomaterial ink containing 12% (*w*/*v*) gelatin, 5% (*w*/*v*) sodium alginate, and 1% (*w*/*v*) carboxymethylcellulose (CMC), phosphate-buffered saline 1× (PBS) solution was first prepared by diluting a concentrated 10× PBS stock solution with ultrapure water.

All precursors were weighed using an analytical balance to the following masses: 6.00 g porcine gelatin, 2.50 g sodium alginate, and 0.50 g CMC. Exactly 50 mL of the prepared 1× PBS was transferred to a 100 mL Berzelius beaker equipped with a magnetic stir bar and placed on a hotplate stirrer DLAB MS-H-280-Pro (DLAB Scientific, Beijing, China) set to 600 rpm and 70 °C. Sodium alginate was slowly added while stirring vigorously to prevent clumping, followed by CMC, and the mixture was maintained at 70 °C for 2 h until both polymers fully dissolved into a viscous solution.

The temperature was then lowered to 36 °C, monitored with a probe thermometer to avoid gelatin denaturation, and the gelatin powder was gradually added under continuous stirring [28]. Additionally, few drops of methylene blue were added for the improvement of contrast for image analysis. After visual confirmation of complete gelatin dissolution into a homogeneous solution, stirring continued at 36 °C for an additional 1 h to ensure full polymer hydration and incorporation. The final biomaterial ink exhibited a physiological pH in the range of 7–8, maintained by the 1× PBS buffer; the addition of biopolymers did not measurably shift the pH under these buffered conditions.

**Figure 1:**
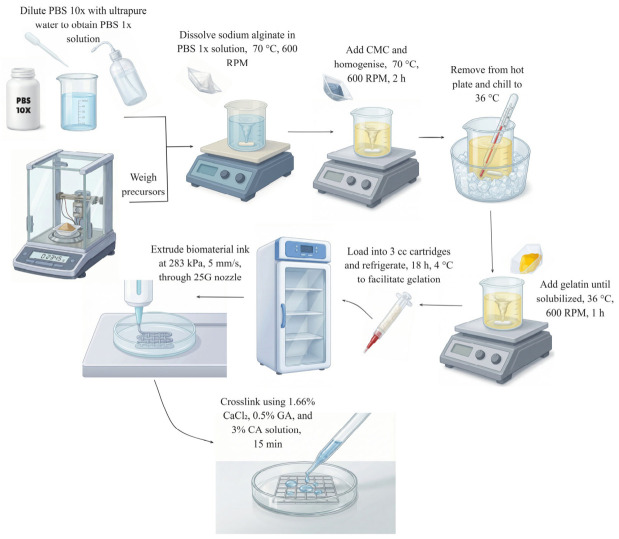
Biomaterial ink synthesis workflow (made using Illustrae [29]).

The resulting biomaterial ink was loaded into 3 cc cartridges for bioprinting and placed in a fridge for 18 h at 4 °C to facilitate gelation, as shown in Figure 1. Subsequent post-printing crosslinked by immersion in a freshly prepared aqueous mixture containing 1.66% CaCl_2_, 0.5% glutaraldehyde, and 3% citric acid for 15 min at room temperature, followed by rinsing in PBS 1 X.

### 2.3. Rheological Characterization

The rheological measurements were conducted using an Anton Paar rheometer (Anton Paar GmbH, Graz, Austria) equipped with a 25 mm plate at 0.5 mm gap and a constant measurement temperature of 25 °C. The sample was characterized by oscillatory tests: (i) strain amplitude sweep and (ii) frequency sweep performed in the linear viscoelastic regime.

### 2.4. Printability Assessment

The printability of a sample can be assessed using a practical filament drop test. This test involves extruding a filament from a higher height to see how the extruded material performs. Ideally, the extruded material should form a steady, smooth filament [12]. If there is no filament produced, but only small subsequent droplets, the material is considered under-gelled (viscosity is too low). On the other hand, over-gelled compositions tend to result in clumps and non-uniform filaments. For this test we used an external syringe pump setup that had an 11-gauge needle as an outlet, which generated an extrusion of 2.4 mm diameter.

### 2.5. Quantitative Print Quality Assessment

This chapter details five specific geometrical quantifications that have become standard for assessing biomaterial ink printability: Collapse Angle (θ), Uniformity Ratio (U), Spreading Ratio (SR), Filament Fusion, Printability Index (P_r_), and Porosity.

#### 2.5.1. Collapse Angle

**Figure 2:**
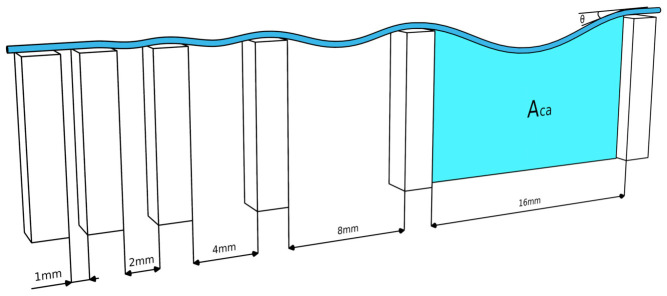
Filament collapse test for assessing self-supporting structural integrity. A biomaterial ink filament is extruded across a platform of pillars with exponentially increasing gap distances (1 mm to 16 mm). The degree of sagging is quantified by the Collapse Angle (θ) at the pillar edges and the Actual Area ($A_{ca}$) beneath the deflected filament. This test evaluates the biomaterial ink’s ability to resist gravitational forces through its inherent yield stress. Standard nozzle size is 22-gauge (0.41 mm).

The Collapse Angle (θ) is a quantitative metric used to evaluate the shape fidelity and mechanical stability of biomaterial ink during 3D printing, specifically through the “filament collapse test” [7]. The filament collapse test assesses a material’s ability to span over gaps without sagging under its own weight. which is crucial for printing overhangs. It involves printing biomaterial inks over a structure with pillars separated by varying, known gap distances, as shown in Figure 2. After the printing process, the extruded biomaterial ink is photographed and digitally analyzed.

The deformation of the filament is quantified using the following metrics:

- Angle of Deflection (θ): The angle formed between the horizontal line of the pillars and the suspended filament at the point where they meet is measured. Larger values indicate greater sagging.
- Rate/Area (*C_f_*): This method calculates the difference between the theoretical rectangular area under a straight filament (*A_ct_*) and the actual area under the sagging filament (*A_ca_*). The collapse rate is expressed as a percentage:

(1) $$C_{f}=\frac{A_{ct}-A_{ca}}{A_{ct}}\times 100\%$$

The test essentially measures the balance between gravitational force and the material’s yield stress and surface tension. A theoretical model relates the deflection angle (θ) directly to the material’s yield stress $\sigma_{0}$

(2) $$\theta =\sin^{-1}\left(\frac{\rho gL}{\sigma_{0}}\right),$$

where

- ρ is the material density;
- g is gravitational acceleration;
- L is half the gap distance between pillars.

If the filament maintains a straight line (low θ value; *C_f_* ≈ 0), the material has high enough yield stress to resist the pull of gravity, indicating good shape retention. On the other hand, significant sagging of the extrusion indicates low yield stress [30], suggesting the material may not be suitable for printing complex overhanging structures.

#### 2.5.2. Uniformity and Spreading Ratios

**Figure 3:**
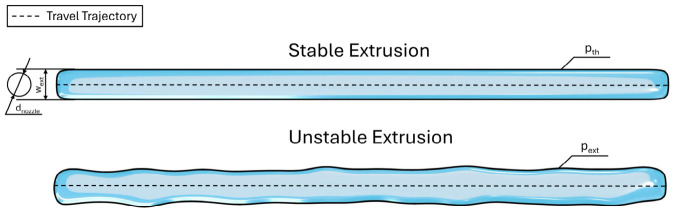
Comparison of filament morphology in extrusion bioprinting. This schematic defines the geometric variables used to quantify deposition precision; Stable Extrusion (**top**): Demonstrates an ideal deposition profile where the filament width $\left(w_{\mathrm{ext}}\right)$ remains consistent relative to the nozzle diameter $\left(d_{\mathrm{nozzle}}\right)$ and follows the travel trajectory with a smooth theoretical perimeter $\left(p_{th}\right)$; Unstable Extrusion (**bottom**): Illustrates the impact of flow fluctuations or pressure lag, resulting in an irregular actual perimeter $\left(p_{\mathrm{ext}}\right)$.

To quantify the morphological characteristics of the extruded filaments, two key metrics are employed: the Uniformity Ratio (UF) and the Spreading Ratio (SR) [31]. The Uniformity Ratio assesses the smoothness and structural consistency of the filament. It is determined by comparing the actual perimeter of the extruded line ($p_{ext}$) to its theoretical perimeter ($p_{th}$), as shown in Figure 3, calculated as:

(3) $$UF=\frac{p_{ext}}{p_{th}},$$

where *p_ext_* is the perimeter of the extruded line and pth is the theoretical perimeter.

The Spreading Ratio is a non-dimensional metric used to evaluate printing precision and resolution. It represents the degree of material deformation upon contact with the substrate and is defined as the ratio of the printed filament width ($w_{ext}$) to the internal diameter of the nozzle ($d_{nozzle}$):

(4) $$SR=\frac{w_{ext}}{d_{nozzle}},$$

where *w_ext_* is the width of the extruded line and *d_nozzle_* is nozzle diameter.

The ideal spreading ratio is 1, which would indicate that the extruded filament is the same width as the printing nozzle. In practice, biomaterial inks such as hydrogels tend to relax and spread after extrusion, meaning the SR is almost always greater than 1.

The experimental procedure involves depositing straight biomaterial ink filaments onto a substrate, followed by high-resolution optical imaging. During the image analysis phase, the captured frames undergo thresholding and segmentation to isolate the extruded geometry from the background. This allows for the digital extraction of geometric properties required for the calculations.

#### 2.5.3. Filament Fusion Test

**Figure 4:**
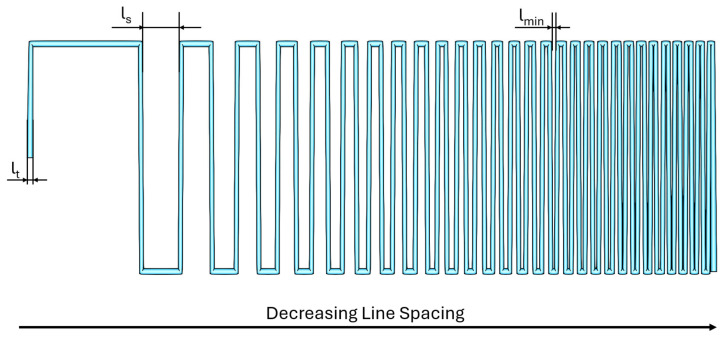
Filament fusion test for resolution limit assessment. A serpentine pattern is printed with decreasing line spacing ($l_{s}$) to identify the threshold of material merging. The test quantifies the interaction between material yield stress and capillary forces. Key parameters include the measured filament thickness ($l_{t}$) and the minimum line spacing ($l_{min}$), which represents the smallest distance achievable before total filament fusion occurs. Ideally, the line thickness should be equal to the nozzle diameter. The line spacing represented in this figure decreases from 6.7 mm to 0.3 mm. Minimum line thickness attainable from this specific test is 0.3 mm (using a 0.4 mm nozzle).

The filament fusion test is a standard method for evaluating the quality of 3D bioprinted constructs. It involves printing a serpentine pattern with varying line spacing to determine the minimum distance at which the lines begin to fuse, as shown in Figure 4 below [32]. This parameter is indicative of the maximum resolution achievable with a specific biomaterial ink and printing system.

The test acts as a practical indicator of the balance between a material’s yield stress and the surface tension (capillary forces) driving deformation. Materials with sufficient yield stress resist the capillary forces trying to minimize surface area, resulting in shorter fused segments and the ability to maintain open pores at smaller distances. Softer materials allow capillary forces to dominate, causing filaments to merge (fuse) over longer distances, decreasing resolution.

Several metrics can be derived from this test: line thickness $(l_{t}$), line spacing $(l_{s}$) and minimum line spacing $(l_{min}$).

**Figure 5:**
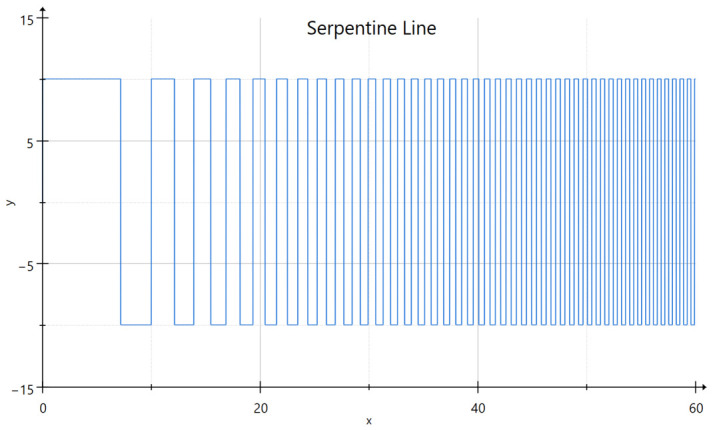
Frequency-modulated serpentine toolpath for filament fusion testing. The trajectory is generated using a square wave function with a modulated frequency (A = 10, F = 0.05, n = 2.1). This approach creates a continuous path with a progressively decreasing period, allowing for the systematic evaluation of the minimum achievable line spacing ($l_{min}$) in a single print operation.

For our experiments, we generated the serpentine line toolpath (Figure 5) using a frequency modulated square wave function, using the following formula:

(5) $$f\left(x\right)=\;A\times sgn\left(10*sin\left(F\;{\times \left|x\right|}^{n}\right)\right),$$

where A is the amplitude of the square wave, F is the base frequency and n is the chirp rate.

To streamline the generation of complex trajectories, we utilized Blender 3D [33] to procedurally design the toolpaths. A custom Geometry Nodes [34] setup was developed, featuring parametric sliders [35] for the various coefficients. This non-destructive workflow allowed for the rapid iteration of print patterns, ensuring that the spatial frequency of the serpentine line could be adjusted dynamically. Following the design phase, the vertex coordinates were extracted and translated into standardized G-code instructions.

#### 2.5.4. Printability Index

The printability index (Pr) is a semi-quantitative metric used to assess the shape fidelity and printing accuracy of biomaterial inks, specifically by evaluating the geometry of pores within a printed lattice or grid structure.

It is mathematically defined based on the relationship between the perimeter (L) and the area (*A*) of the pore:

(6) $$Pr=\frac{L^{2}}{16\times A}$$

**Figure 6:**
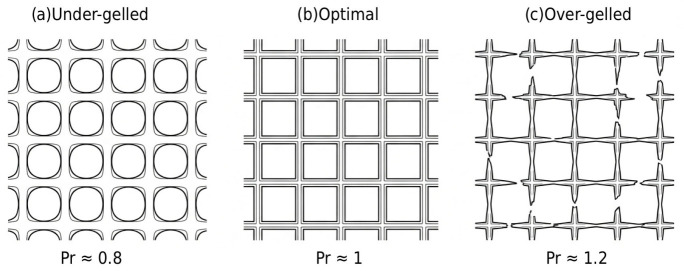
Morphological assessment of 2D grid pores using the Printability Index (Pr). (**a**) Under-gelled regime (Pr < 1): Low viscosity leads to filament spreading and rounded pore corners. (**b**) Optimal regime (Pr~1): High fidelity results in well-defined, square pore geometry. (**c**) Over-gelled regime (Pr > 1): Excessive yield stress causes irregular extrusion and jagged filament boundaries, resulting in complex pore shapes.

A Pr value of 1 means that a pore is perfectly square. Values lower than 1 indicate rounded pores. This is typically a sign of filament fusion (merging), material spreading, or low viscosity (Figure 6). Pr values higher than 1 indicate an irregular or complex pore shape. This is often associated with non-uniform extrusion of filament, potentially caused by over-gelation or excessive yield stress.

For the printability index assessment, 2D grid patterns were procedurally generated within Blender 3D (https://www.blender.org/). Utilizing a Geometry Nodes setup, we implemented parametric controls to adjust grid density and line spacing. The resulting toolpaths were subsequently post-processed into G-code for execution on both extrusion systems.

#### 2.5.5. Porosity Evaluation Through Image Analysis

This binarization and particle-analysis workflow follows established methodologies for quantifying shape fidelity [7] and pore architecture [12] in extrusion-based bioprinting, and has also been employed for 2D optical porosity assessment of biomaterial ink scaffolds in analogous studies [36].

This digitally evaluated porosity was then directly compared with the theoretical porosity derived from the CAD design, enabling a quantitative assessment of how closely the printed constructs matched the intended pore network.

**Figure 7:**
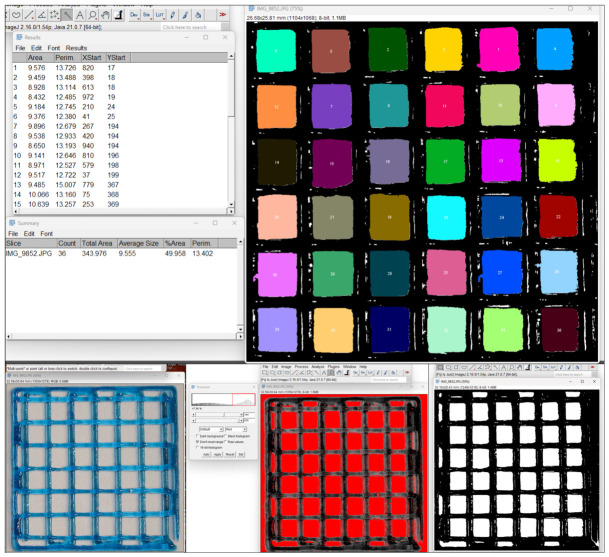
Image analysis workflow (screen captures from Fiji, https://fiji.sc/).

Raw images of the printed grids were acquired directly after printing using a Canon R6 mirrorless camera at a resolution of 3648 × 5472 pixels, with the lens set to 73 mm and uniform illumination from above. Pore geometry and scaffold porosity were quantified from these images using Fiji (Figure 7) [37], which were first converted to 8-bit grayscale and the contrast was adjusted to maximize the separation between the biomaterial ink filaments and the background. A global threshold was then applied using the Threshold tool to obtain a binary image, in which the pores appeared as bright regions and the strands as dark boundaries. On this binarized image, the ‘Analyze Particles’ [36] function was used with a size range set from 2.5 pixel^2^ to infinity and a circularity range of 0.00–0.10, in order to selectively segment the 36 square pores and exclude small artefacts or partially captured features at the image borders. This procedure generated a mask covering the entire scaffold area and returned, for each pore, its projected area and perimeter, used to compute the hydraulic radius (area/perimeter) as a compact descriptor of pore size.

For each image, Fiji also provided a summary of the total pore area expressed as a percentage of the analysed field of view, which was used as the evaluated porosity of the scaffold and directly compared with the theoretical porosity calculated from the design.

## 3. Results

### 3.1. Rheological Characterization

**Figure 8:**
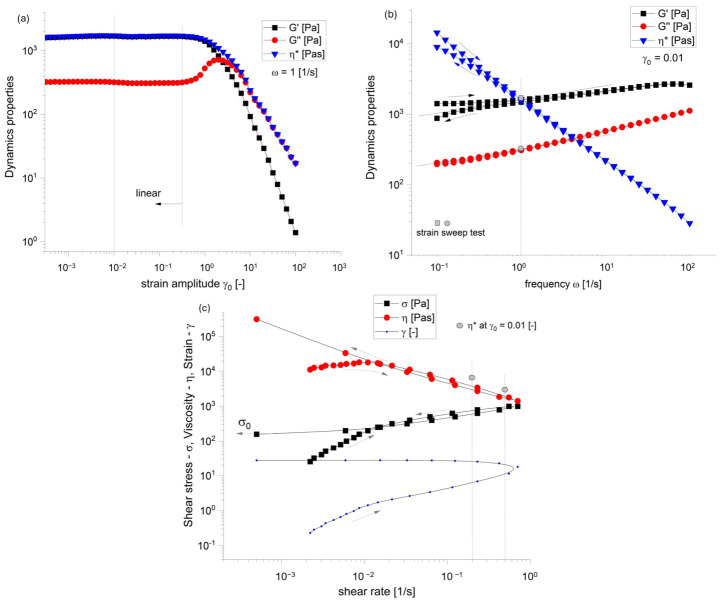
Viscoelastic profile of the biomaterial ink in oscillation tests: (**a**) strain amplitude sweep, (**b**) frequency sweep. The persistent relationship where G′ > G″ across the entire measured domain highlights the biomaterial’s gel-like nature at small frequencies. Furthermore, the significant decrease in η* as ω increases confirms the material’s shear-thinning behaviour nature, which facilitates the precision and resolution observed in the screw-driven volumetric extrusion samples. The high value of viscosity recorded at ω = 0.1 [1/s] suggests the existence of the yield stress in the limit of zero shear rate. (**c**) Flow curve—10 s/point in the interval of shear rate $\dot{\gamma }\in$ (10^−4^ ÷ 1) [1/s] (up and down). The data at $\dot{\gamma }<0.01\;[1/s]$ is not in steady state.

The oscillatory strain amplitude sweep test, Figure 8a, evidences the dominated elastic behavior in the linear regime (SAOS), G′ > G″ at $\gamma_{0}<0.3\;\left[-\right],$ followed by a non-monotonicity of loss/viscous modulus G″ in MAOS regime. The local thickening of G″ is a rheological characteristic of composite viscoelastic materials, like soft solids, creams and pastes [30]. The elastic/storage modulus (G′) dominates G″ in the frequency oscillatory test performed at $\gamma_{0}<0.01\;\left[-\right],$ Figure 8b. The gel-like behavior with G′ > G″ is observed at small frequencies ω, where both moduli decrease almost parallel for ω<5 [1/s]. The ink exhibits a pronounced shear-thinning nature, characterized by a sharp decrease in complex viscosity as the angular frequency increases. This property is required for extrusion-based bioprinting as it ensures the material flows easily through the fine nozzle under high-shear conditions while recovering its high-viscosity state almost instantly upon deposition to “lock in” the printed architecture. The presence of yield stress $\sigma_{0}≅350\;Pa$ is observable in the flow curve shown in Figure 8c.

### 3.2. Printability Assessment

**Figure 9:**
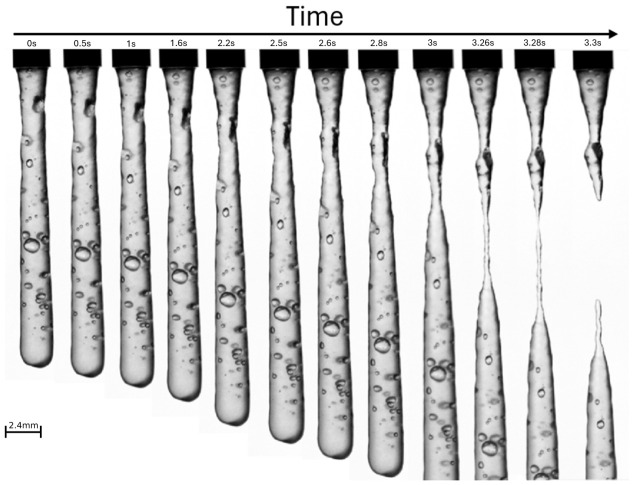
High-speed temporal analysis of the filament drop test. This sequence, captured with a Phantom high-speed camera, illustrates the morphological evolution of the biomaterial ink as it is extruded through an 11-gauge needle. Initially, the material forms a stable, continuous column where internal cohesive forces balance the weight of the ink. As the strand length increases, the gravitational load induces uniaxial stretching, leading to necking (localized thinning). The high-speed footage identifies the precise moment of filament fracture, defining the critical limit where the material’s elastic forces are overcome by gravity. This confirms the biomaterial ink is ready for continuous extrusion bioprinting.

To establish a baseline for printability, we first assessed whether the biomaterial ink formulation could sustain a stable extrusion profile across both hardware platforms. This was evaluated via a filament drop test, as illustrated in Figure 9, where the ink was extruded from an elevated height to observe the transition from initial droplet formation to a continuous, cohesive fiber. This phenomenon is governed by a dynamic balance between gravitational forces, pulling the material downward, and the material’s yield stress and viscoelasticity, which resist thinning and fracture. It is important to note that this specific test was conducted using a larger 11-gauge needle to facilitate the observation of the weight-elastic force balance. Subsequent benchmarking prints utilized a significantly finer 22-gauge needle. Successful formation of a non-breaking filament confirms that the cohesive properties of the formulation are sufficient to overcome gravitational stretching, ensuring the material can be reliably deposited as a continuous strand without premature breakage. The successful continuous filament in this test shows a material possessing a yield stress and elastic modulus high enough to overcome both the downward pull of gravity and the pinching effect of surface tension.

#### 3.2.1. Collapse Angle

This evaluation measures the deflection of a filament suspended between pillars of increasing distances, providing insight into the material’s yield stress and its ability to resist gravitational forces immediately post-extrusion.

**Figure 10:**
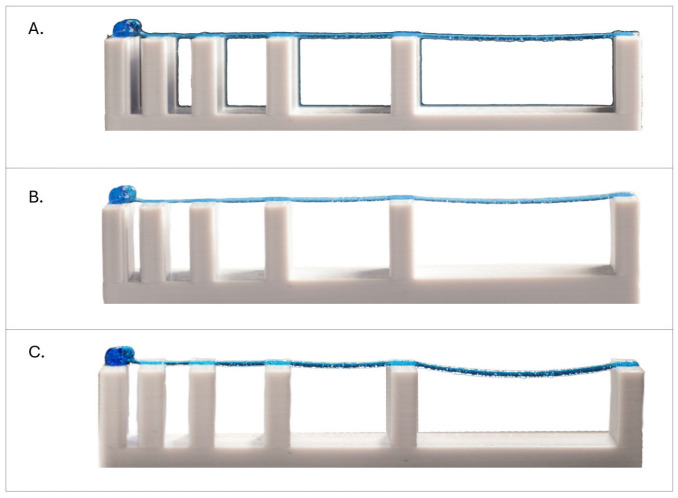
Baseline consistency of filament collapse tests. Three identical replicates (**A**–**C**) demonstrate the biomaterial ink extruded across varying pillar gap distances ranging from 1 mm to 16 mm. The material possessed sufficient yield stress to bridge all gaps. The uniform morphology across all three trials indicates high process consistency from the screw-driven extruder. Deflection angle was too small to be measured.

Initially, the baseline consistency of the platform and material was evaluated. Three replicate samples were printed onto a customized fixture featuring gap distances ranging from 1 mm to 16 mm (Figure 4). As illustrated in Figure 10, the biomaterial ink demonstrated sufficient yield stress to successfully bridge all spans without rupture. Furthermore, the visual uniformity across the three trials (A, B, and C) confirmed the high repeatability of the screw-driven extrusion process under constant parameters.

Following baseline validation, a second experiment was performed to characterize the influence of volumetric flow rate on structural stability. Using the maximum span distance of 16 mm, filaments were deposited at three distinct flow rates: 50%, 75%, and 100% of the theoretical output.

**Figure 11:**
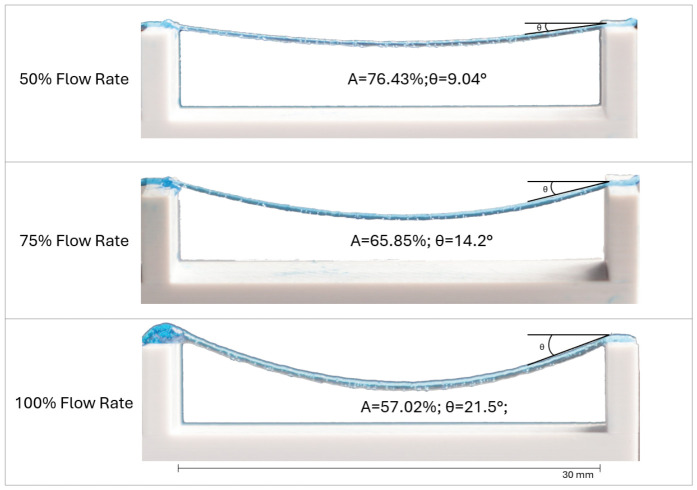
Influence of flow rate modulation on filament deflection. A comparison of filament sagging across a fixed 32 mm span at three different commanded flow rates. (**Top**) (50% Flow Rate): The reduced deposited volume results in the lowest filament mass, yielding the highest stability with a minimal deflection angle of θ = 9.04°. (**Middle**) (75% Flow Rate): Intermediate mass results in moderate deflection at θ = 14.2°. (**Bottom**) (100% Flow Rate): The full volumetric output creates the heaviest filament, inducing the highest gravitational stress and the largest deflection angle of θ = 21.5°. Calculated apparent yield stress values are 428 Pa for the 100% sample, 640 Pa for the 75% sample and 998 Pa for the 50% sample.

The results, presented in Figure 11, demonstrate a direct correlation between flow rate and filament sagging. While the intrinsic material yield stress remains constant, increasing the flow rate significantly increases the linear mass (and thus the effective gravitational load) of the extruded filament. At a 50% flow rate, the reduced mass of the filament resulted in minimal sagging, exhibiting a deflection angle of only 9.04°. Increasing the flow to 75% raised the angle to 14.2°. Finally, at a 100% flow rate, the heaviest filament experienced the greatest gravitational stress, resulting in the maximum observed deflection angle of 21.5°.

We also calculated the Collapse Rate ($C_{f}$) for each filament. Naturally, as the flow rate is increased and thicker strands are produced, the $C_{f}$ linearly decreases.

The calculated yield stress exhibited a range between 428 Pa and 998 Pa, in the range of the measured yield stress $\sigma_{0}≅350\;Pa.$ The classical formula used in bioprinting literature is derived from a simplified force balance. In this model, the filament is treated as a one-dimensional string or a beam where the weight is assumed to be a point load or a uniform linear load that does not vary with the diameter. Because the standard formula does not adjust for the actual volume of material deposited, the resulting value is an apparent yield stress.

This test highlights a critical advantage of the screw-driven system: the ability to actively modulate structural stability through precise volumetric control. By “under-extruding”, the gravitational load on suspended features is minimized, allowing the material to span greater distances with significantly higher fidelity than possible at full volumetric output. This demonstrates that the system does not merely rely on material properties alone but provides a tunable parameter to engineer the structural limits of the scaffold.

#### 3.2.2. Line Tests

To further quantify the precision of the deposition process, straight-line filaments were printed and analyzed optically to determine their Uniformity and Spreading Ratios. These metrics are useful for assessing whether the extrusion system can maintain a consistent strand diameter and how effectively it can control the lateral expansion of the biomaterial ink.

**Figure 12:**
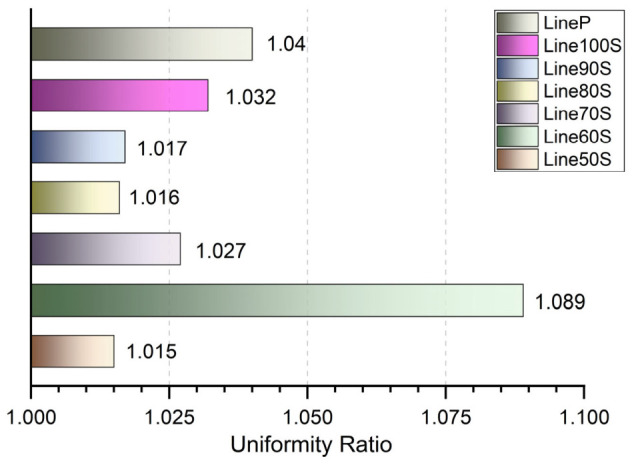
Quantitative evaluation of filament Uniformity Ratio. This bar chart displays the uniformity ratio for the pneumatic system (LineP) and the screw extruder at varying flow rates (Line50S to Line100S). All samples show a ratio approaching 1.0, indicating that both extrusion technologies provide highly consistent filament diameters throughout the deposition process.

The Uniformity Ratio serves as a measure of the diameter consistency along the length of a single filament. As shown in Figure 12, both the pneumatic and screw-driven systems exhibited excellent uniformity, with all measured values remaining close to the ideal ratio of 1.0. The pneumatic system achieved a ratio of 1.04, while the screw-driven samples across all flow rates ranged between 1.015 and 1.089. This high degree of consistency across technologies suggests that once flow is stabilized, both systems can produce filaments with minimal fluctuations in width.

**Figure 13:**
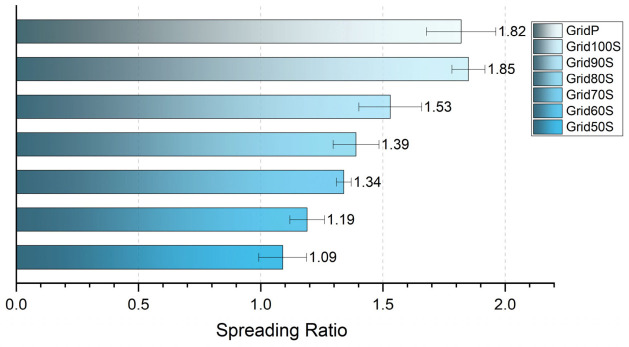
Impact of flow rate on the Spreading Ratio (SR). The chart illustrates the linear relationship between the commanded flow rate and the resulting spreading of the filament. While the baseline pneumatic (GridP) and 100% screw (Grid100S) samples show significant expansion (SR~1.8), the 50% flow rate sample (Grid50S) achieves a ratio of 1.09. This confirms that volumetric modulation can be used to compensate for die swell and print filaments that match the physical dimensions of the nozzle.

The Spreading Ratio revealed a much more significant distinction between the extrusion settings. As illustrated in Figure 13, the pneumatic system produced a substantial spreading effect with an SR of 1.82. Similarly, the screw extruder at a 100% flow rate resulted in an SR of 1.85, indicating that the material naturally expands by nearly 85% due to the die swell effect.

However, the screw extruder’s volumetric control allowed for a systematic reduction in this expansion. We observed a linear decrease in the spreading ratio as the flow rate was reduced from 100% down to 50%. Notably, at a 50% flow rate, the spreading ratio dropped to 1.09, meaning the final line thickness almost perfectly matched the nozzle diameter. This demonstrates that the screw-driven system can effectively combat die swell by “under-extruding” the material, providing a unique operational parameter to achieve high-resolution prints that are otherwise limited by the rheological properties of the biomaterial ink.

### 3.3. Filament Fusion Tests

To quantify the resolution limits of both platforms, a filament fusion test was conducted using a frequency-modulated serpentine toolpath with a programmed line spacing (ls) decreasing from 6.7 mm down to 0.2 mm. All samples were deposited at a constant print speed of 20 mm/s. For the pneumatic system, an extrusion pressure of 75 kPa was applied, the minimum threshold required to initiate flow. For the screw-driven system, the flow rate was dynamically modulated as a function of travel speed and theoretical volume (10 mm^3^/rev calibration), utilizing its positive displacement capabilities.

**Figure 14:**
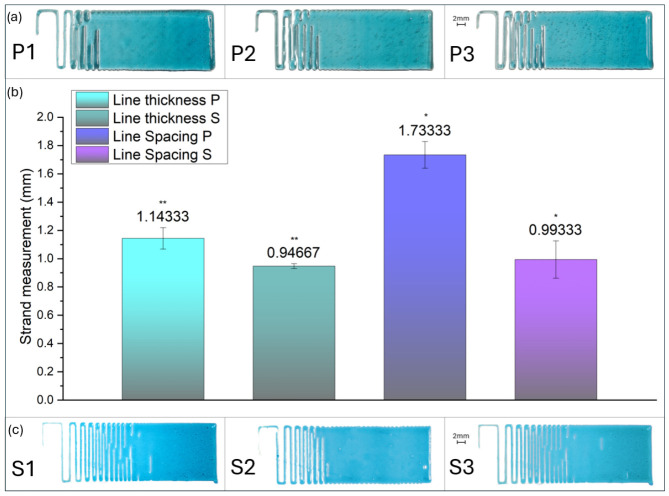
Comparison of filament fusion and spatial resolution. (**a**) Representative serpentine patterns from the pneumatic system showing early filament coalescence. (**b**) Statistical analysis of line thickness and minimum spacing between the two systems. The screw-driven system (S) achieves a significantly smaller lmin (0.99 mm) and more refined lt (0.95 mm) than the pneumatic system (P). Error bars represent standard deviation (mean ± SD, *n* = 3). Asterisks indicate statistically significant differences relative to GridP (two-sample *t*-test, * *p* < 0.05, ** *p* < 0.01). (**c**) Screw-driven samples demonstrating superior spacing maintenance and sharp-stop precision.

As illustrated in Figure 14, the screw extruder demonstrated an advantage in both resolution and dimensional consistency. Quantitatively, the Minimum Line Spacing (lmin)—the threshold before adjacent filaments coalesce was significantly lower for the screw system (0.99 mm) compared to the pneumatic system (1.73 mm). Furthermore, the screw-driven filaments exhibited a mean Line Thickness (lt) of 0.95 mm, achieving a 17% reduction in strand width compared to the pneumatic mean of 1.14 mm. For the quantitative assessment of resolution, the minimum line spacing was defined as the final completely open gap before the onset of coalescence. Under this criterion, the pneumatic system performed poorly, as inherent flow instabilities led to early filament fusion across wider spacings.

The reduction in line thickness achieved by the screw-driven system suggests a significant decrease in the die swell effect compared to the pneumatic setup. In pneumatic extrusion, the compressed air acts as a constant energy source, maintaining high stress on the fluid even during subtle travel speed changes. This leads to ‘over-swelling’ at the nozzle exit. Conversely, the screw pump’s mechanical dosing reduces the pressure fluctuations, resulting in a more relaxed extrudate with a lower spreading ratio and, consequently, finer spatial resolution.

The screw-driven system’s primary mechanical advantage lies in its ability to mitigate the die swell by modulating the volumetric output independently of the travel speed. As viscoelastic biomaterial inks exit the nozzle, they naturally expand as internal stresses relax. However, the positive displacement nature of the screw pump allows for under-sampling the theoretical flow rate to compensate for this expansion.

**Figure 15:**
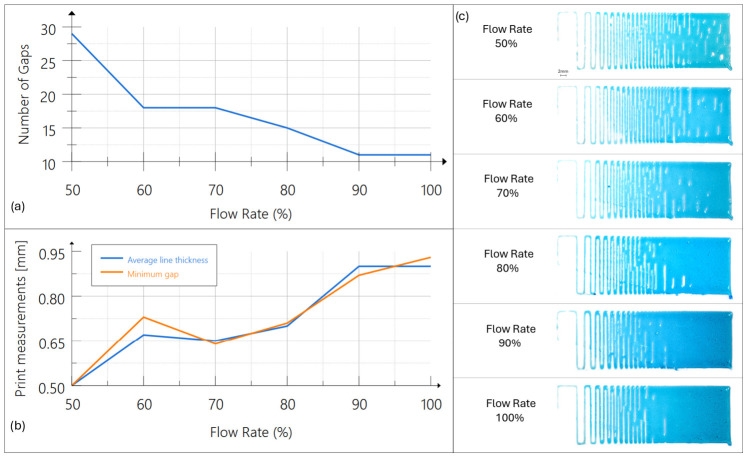
Impact of flow rate modulation on spatial resolution and die swell compensation. (**a**) Graph showing the increasing number of open gaps as the flow rate is reduced from 100% to 50%. (**b**) Reduction in average line thickness and minimum gap width as a function of decreased volumetric output. (**c**) Visual comparison of serpentine samples, demonstrating the transition from fused filaments at high flow rates to high-resolution, distinct strands at lower flow rates.

As illustrated in Figure 15, more tests were performed by decreasing the flow rate from 100% down to 50% of the theoretical volume while maintaining a constant print speed. The results reveal a clear correlation between the flow rate and the resulting print resolution.

The screw-driven system demonstrates a significant technical advantage over the pneumatic counterpart by facilitating a constant, volumetrically controlled output even at reduced flow volumes. In pneumatic extrusion, reducing the applied pressure below a critical threshold frequently results in flow interruption or stuttering because of the material’s inherent yield stress [38]. Conversely, by utilizing under-extrusion, the screw pump accommodates the phenomenon of die swell without exceeding the target filament width. This capability provides a tunable operational parameter that allows the creation of parts with varying levels of detail, depending on the printed feature [4].

### 3.4. Grid Tests

**Figure 16:**
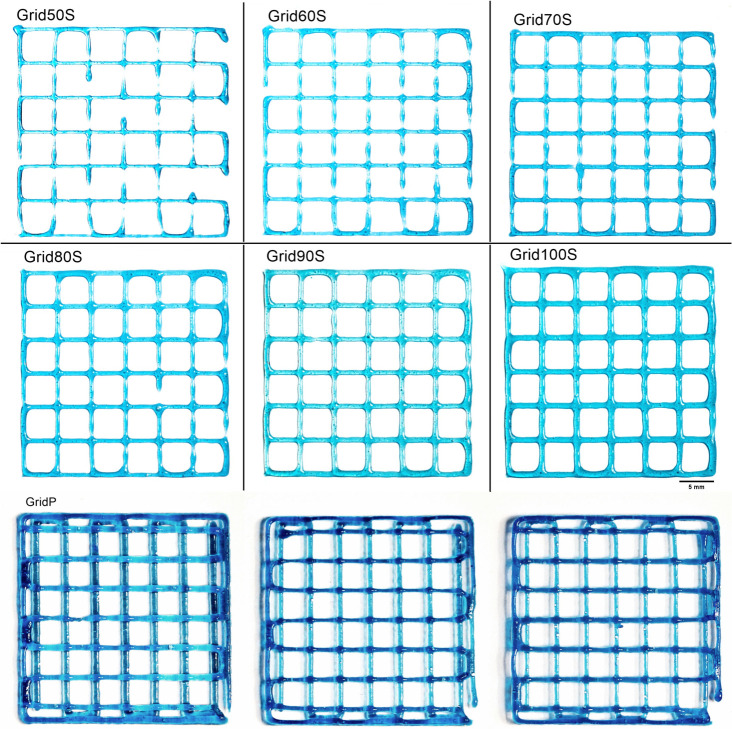
Qualitative comparison of grid constructs. Visual overview of grids printed using the screw extruder at varying flow rates 50% to 100%) alongside the pneumatic system (GridP). Note the transition from thin, occasionally discontinuous strands at 50, 60 and 70% flow to the dense, over-gelled appearance of the pneumatic sample. Gel accumulation at the intersections is visible across all samples due to surface tension.

To evaluate the transition from individual filaments to complex three-dimensional architectures, grid structures 30 × 30 mm featuring 36 individual 5 mm pores were printed, as shown in Figure 16. These constructions consisted of two layers with a fixed layer height of 0.51 mm and a print speed of 20 mm/s. This test serves as a real-world scenario to assess how volumetric control influences pore geometry and structural stability in multi-layer prints.

A critical observation in the lower flow rate samples (Grid50S and Grid60S) was the presence of filament discontinuities. This is attributed to the high layer height (0.51 mm) relative to the extruded strand thickness (0.45 mm) and 0.49 mm, respectively. When the layer height exceeds the filament diameter, the extruded material lacks sufficient contact with the previous layer or the substrate to anchor properly. This leads to the strand snapping or pulling thin across the open spans. While this is easily corrected by matching the layer height to the strand diameter, we maintained a constant 0.51 mm height across all tests to ensure the data remained comparable across the different volumetric settings.

In the crossover regions, where two filaments intersect, we observed a localized accumulation of gel. This is a result of surface tension and wetting [25]; as the fresh extrudate crosses an existing filament, the two viscoelastic masses tend to coalesce to minimize surface energy. This effect is most pronounced in the high-flow samples (Grid100S and GridP), where the larger material volume leads to “blurred” corners and rounded pores, lowering the Printability Index. Conversely, the lower flow rates maintained sharper corner definitions, even with the discontinuities, proving that volumetric modulation is the primary tool for enabling pore squareness.

**Figure 17:**
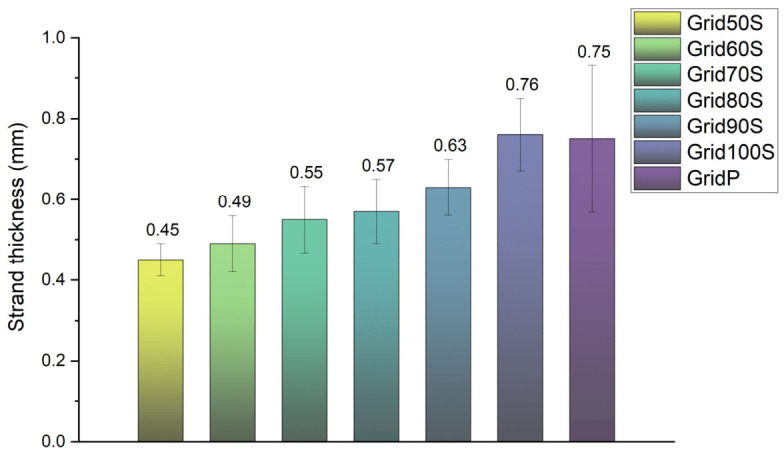
Statistical distribution of strand thickness across grid samples. This bar chart quantifies the average strand thickness for each print setting. The data highlights the screw extruder’s capacity for fine-tuning resolution, showing a clear linear progression from 0.45 mm up to 0.76 mm, whereas the pneumatic system is restricted to a wider 0.75 mm baseline.

As illustrated in Figure 17, there is a linear relationship between the commanded flow rate and the resulting strand thickness. At 100% flow, the screw extruder produced a strand thickness of 0.76 mm, which is nearly identical to the pneumatic system’s output of 0.75 mm. However, by utilizing the screw extruder’s ability to “under-extrude,” we successfully reduced the strand thickness to 0.45 mm at a 50% flow rate—a 40% reduction in width compared to the pneumatic baseline.

#### 3.4.1. Hydraulic Radius

**Figure 18:**
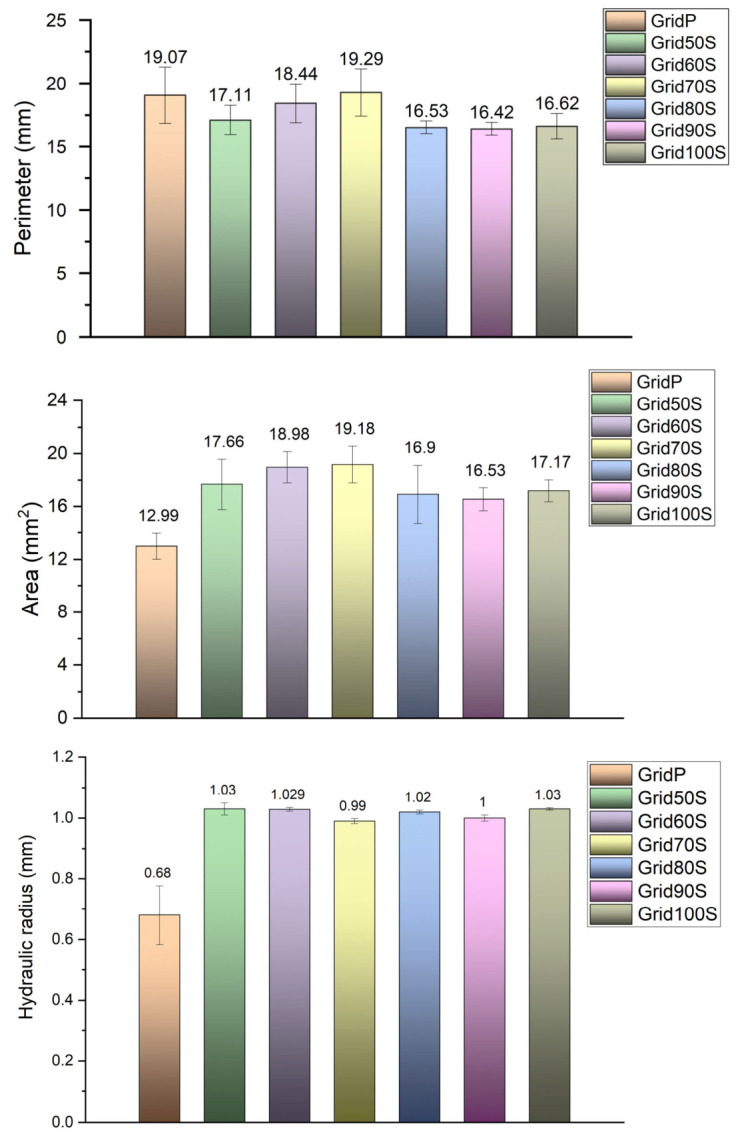
Histograms of pore hydraulic radius, perimeter and area across the 3D printed grids.

The pore-level metrics derived from image analysis further quantified how extrusion mode and volumetric factor shaped the internal scaffold architecture, as illustrated in Figure 18. The hydraulic radius, defined as the ratio between pore area and perimeter, was markedly lower for the pneumatically printed GridP (0.68 mm) than for all screw-driven grids, which clustered around 1.0–1.03 mm, indicating that pneumatic extrusion produced more constricted pores, whereas volumetric control maintained a characteristic pore size close to the design target. Consistently, GridP also exhibited the largest average perimeter (19.07 mm) and the smallest pore area (12.99 mm^2^), reflecting elongated, irregular pores formed by swollen and partially fused filaments. In contrast, the screw-driven samples, particularly Grid50S–Grid70S, showed shorter perimeters (17.11–19.29 mm) combined with larger areas (17.66–19.18 mm^2^), characteristic of more regular, near-square pores. These combined descriptors corroborate that the screw pump affords tighter control over pore size and shape than the pneumatic system [21], especially in the intermediate extrusion range where hydraulic radius, perimeter and area converge toward the intended grid geometry.

#### 3.4.2. Printability Index

To evaluate the geometric fidelity and structural integrity of the printed constructs, we utilized the Printability Index as standardized metric that quantifies the degree of “squareness” in printed pores.

**Figure 19:**
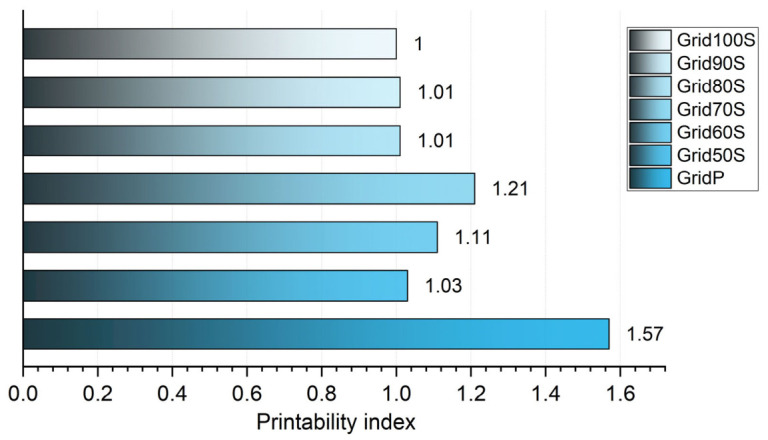
Evaluation of the Printability Index (Pr). The chart displays the Pr values for each grid, where a value of 1.0 indicates a perfect square pore. The Grid100S sample achieved an ideal 1.0, whereas the pneumatic sample (GridP) reached 1.57, indicating significant pore rounding and loss of geometric fidelity.

The Pr provided an additional quantitative measure of how closely the printed pores matched the intended square geometry. GridP showed the highest value (Pr = 1.57), indicating strongly distorted pores with elongated, irregular boundaries, consistent with its low porosity and large perimeter (Figure 19). In contrast, all screw-driven grids clustered near the ideal value of 1, with Pr ranging from 1.03 for Grid50S to 1.00 for Grid100S, indicating only limited deviations from square pores across the tested extrusion factors. Intermediate conditions (Grid70S–Grid80S, Pr = 1.01) lay closest to unity, suggesting that they offer the most favourable balance between pore regularity and continuous filament formation within the evaluated parameter window.

#### 3.4.3. Porosity Assessment

The porosity assessment measures the void fraction, or the total open space left inside the printed grid. From a cell flow perspective, these voids act as the channels for nutrient transport and cell movement. If the ink spreads too much due to die swell, it clogs these channels and reduces the available volume for fluid to pass through the scaffold.

**Figure 20:**
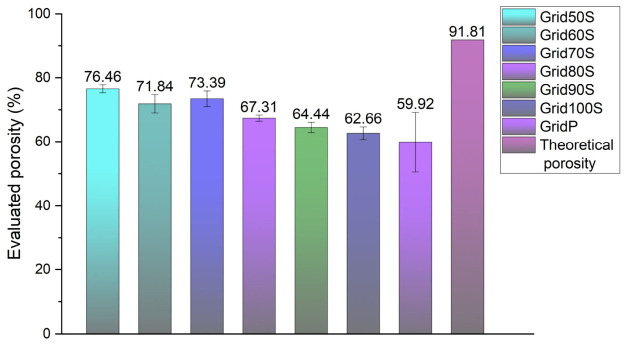
Statistical evaluation of scaffold porosity. This bar chart compares the experimental porosity of the grids against the theoretical design limit of 91.81%. The data shows that lower flow rates (Grid50S) preserve more void space than the pneumatic (GridP) or full-volume (Grid100S) settings. The error bars indicate the consistency of pore maintenance across the 30 × 30 mm grid area.

The porosity analysis illustrated in Figure 20 showed that all printed grids exhibited lower open volume than the theoretical design porosity of 91.81%, even though they were extruded through a 410 µm nozzle, indicating that viscoelastic die swell and post-deposition spreading partially occluded the pores. Among the screw-driven samples, Grid50S achieved the highest evaluated porosity (76.46%), followed by Grid70S (73.39%) and Grid60S (71.84%), showing that reduced extrusion factors favour more open and well-resolved pore networks. As the extrusion factor increased from 80% to 100%, porosity progressively decreased from 67.31% (Grid80S) and 64.44% (Grid90S) to 62.66% (Grid100S), reflecting increasing overfilling of the lattice. GridP, printed pneumatically at constant pressure, showed the lowest porosity (59.92%), consistent with its higher spreading ratio and thicker strands and highlighting the limited ability of pneumatic extrusion to preserve the designed pore volume compared with volumetrically controlled screw-driven printing.

## 4. Discussion

The results show that screw-driven volumetric extrusion provides more precise control over deposited biomaterial ink volume and scaffold architecture than pneumatic pressure-driven extrusion, in line with prior reports on positive-displacement pumps in bioprinting. In the long-line and grid benchmarks, adjusting the extrusion factor primarily modulated filament width and pore openness, while the uniformity ratio remained close to one, indicating that the screw mechanism did not introduce additional flow instabilities. By contrast, the pneumatic configuration produced thicker, more swollen filaments with higher spreading ratios and lower porosity, consistent with pressure overshoot and hysteresis described for air-driven systems [4].

In practical terms, the screw-driven volumetric control enabled us to reliably resolve grid pores and serpentine features in the 200–400 μm range, closely matching the 410 μm nozzle diameter and preserving sharp pore corners at the sub-millimetre scale. This sub-millimetre stability is critical for maintaining open channels and well-defined pore geometries in biomaterial ink scaffolds intended for tissue engineering applications.

Our quantitative metrics align with prior work on shape fidelity tests for extrusion bioprinting, which highlight yield stress and viscoelastic recovery as key determinants of pore geometry [12] and filament stability [7]. In agreement with these studies, the screw-driven system’s ability to under-extrude (50–70% extrusion factor) effectively mitigated die swell and spreading, reducing SR towards 1.0 while preserving square pores (Pr ≈ 1.0). Conversely, the pneumatic system—despite operating at the minimum extrusion pressure—produced rounded and partially fused pores (Pr > 1, SR ≫ 1), underscoring the impact of air compressibility and hysteresis on flow control. Our data therefore support the view that positive displacement extrusion can decouple the material’s complex rheology from the deposited geometry, enabling finer control over internal porosity [9] and micro-channel connectivity than pressure-driven systems [12].

From the perspective of our initial hypothesis—that volumetric control can compensate die swell and improve shape fidelity without changing nozzle or ink formulation—the data support a broad tunable window between 60% and 100% extrusion factor, where pore geometry approaches the targeted square morphology and the printability index remains near unity. The poorer performance observed at 50–80% extrusion (strand discontinuities, local under-filling) is best interpreted as a process-integration artefact: the layer height was kept at 0.51 mm, a value appropriate for 90–100% flow, so the reduced filament cross-section was insufficiently compressed to ensure continuous bonding. This suggests that volumetric under-extrusion must be coupled to a proportionally lower layer height, a point that is rarely made explicit in earlier grid-based printability studies but is critical for exploiting screw-driven systems to their full potential.

Placed in the broader context of bioprinting quality metrics, these findings reinforce that classical 2D descriptors (spreading ratio, printability index) remain informative when systematically linked to controllable hardware parameters rather than only to biomaterial ink rheology. For applications requiring precise porosity and perfusable channels [39]—such as cartilage [40] or vascularized soft-tissue models [22]—our results imply that screw-driven extruders can deliver finer spatial resolution and better pore interconnectivity than pneumatic platforms at comparable viscosities, provided that slicing parameters are co-optimized. Future work should (i) implement coupled optimization of volumetric flow and layer height, (ii) extend the analysis to cell-laden inks to assess how viable cell loading interacts with the identified parameter window, and (iii) compare additional nozzle diameters and grid frequencies to generalize the proposed workflow into a transferable calibration protocol for volumetric bioprinting systems.

## 5. Conclusions

This study demonstrates that screw-driven volumetric extrusion provides significantly better control over filament morphology and scaffold porosity than conventional pneumatic pressure-driven bioprinting when using the same nozzle and biomaterial ink formulation. By systematically varying the extrusion factor from 50% to 100%, we showed that the screw pump can decouple line width from travel speed and recover resolution lost to viscoelastic die swell, enabling pore geometries and printability indices that remain close to the theoretical design over a broad operating window. In contrast, the pneumatic system produced thicker, more swollen strands with higher spreading ratios and consistently lower porosity, confirming long-standing concerns about pressure hysteresis and limited fine control in air-driven setups.

The occurrence of strand discontinuities and local under-filling at low extrusion factors highlights an important process-integration insight: volumetric under-extrusion cannot be tuned in isolation but must be accompanied by proportional adjustments of layer height to maintain adequate filament compression and interlayer bonding. This coupling between hardware-level dosing and slicing parameters is rarely addressed explicitly, yet it is essential for translating volumetric control into reliable improvements in grid fidelity, especially for scaffolds targeting perfusable pore networks [41,42].

This work presents several limitations that should be acknowledged. First, all benchmarking was performed using a single biomaterial ink formulation (12% gelatin/5% sodium alginate/1% CMC); the generality of the observed trends should be validated for biomaterial inks with different rheological signatures. Second, this study focused on acellular constructs; future work will confirm whether the same volumetric control strategies preserve cell viability and function in cell-laden bioinks. Third, only one commercial pneumatic platform and one custom screw-driven extruder were evaluated, limiting direct generalization to other hardware configurations.

Despite these constraints, the presented benchmarking workflow offers a quantitative framework that can be transferred to other biomaterial inks and extrusion platforms, supporting rapid calibration of new systems and more rational selection of process windows for cell-laden constructs [43]. Ongoing work now focuses on extending this approach by integrating a flow sensor into the screw pump assembly, which will enable real-time monitoring and feedback control of the volumetric flow rate. This addition is expected to enhance dosing precision, improve correlation between commanded and actual extrusion volumes, and facilitate more accurate translation of volumetric data into printable design parameters.

## Figures and Tables

**Table 1:** Sample denominator and 3D printing parameters used.

Sample Name	Number of Prints	System Used	Printing Speed	Printing Pressure/ Extrusion Factor	Nozzle Diameter	Printing Temperature
GridP	3	Cellink BIO X	11 mm/s	75 kPa	22 G (410 μm)	25 °C
Serpentine P1, P2, P3			20 mm/s			
Grid50S		Screw pump extruder	20 mm/s	50%		
Grid60S				60%		
Grid70S				70%		
Grid80S				80%		
Grid90S				90%		
Grid100S				100%		
Serpentine SF50				50%		
Serpentine SF60				60%		
Serpentine SF70				70%		
Serpentine SF80				80%		
Serpentine SF90				90%		
Serpentine S1, S2, S3				100%		

Note: For the pneumatic system, printing parameters are defined by the applied air pressure (kPa), as the platform does not provide direct flow-rate feedback. For the screw-driven system, parameters are defined by the volumetric extrusion factor (%), leveraging its positive displacement mechanism to prescribe the extruded volume per unit travel distance.

## Data Availability

The raw data supporting the conclusions of this article will be made available by the authors on request.

## Abbreviations

The following abbreviations are used in this manuscript:

2D	Bidimensional
3D	Tridimensional
CT	Computed tomography
MRI	Magnetic resonance imaging
DIW	Direct Ink Writing
FDM	Fused Deposition Modelling
Pr	Printability index
UF	Uniformity Ratio
SR	Spreadability Ratio
lmin	Minimum Line Spacing
CMC	Carboxymethylcellulose
PBS	Phosphate-buffered Saline
CaCl_2_	Calcium chloride
